# Depicting the Immunological Landscape of Basal Cell Carcinoma Subtypes

**DOI:** 10.1111/cup.70150

**Published:** 2026-06-09

**Authors:** Isabella Dommann, Gaetana Restivo, Isabel Kolm, Federica Sella, Patrick Turko, Phil F. Cheng, Mitchell Levesque, Corina Dommann‐Scherrer, Reinhard Dummer, Egle Ramelyte

**Affiliations:** ^1^ Department of Dermatology University Hospital Zurich and Faculty of Medicine, University of Zurich Zurich Switzerland; ^2^ Department of Dermatology Cantonal Hospital Lucerne Lucerne Switzerland; ^3^ Department of Oncology Geneva University Hospital Geneva Switzerland; ^4^ Department of Pathology Cantonal Hospital Winterthur Winterthur Switzerland

**Keywords:** BCC, HLA class‐I/II, immunotherapy, morphological subtypes, PD‐L1

## Abstract

**Background:**

Anti‐PD‐1 response has been associated with higher PD‐1 ligand (PD‐L1) expression levels in different cancer types. Cemiplimab, an anti‐PD‐1 antibody, has been shown to induce a response rate of 31% and 22% in patients with locally advanced basal cell carcinoma (BCC) and metastatic BCC respectively, who have progressed or are intolerant to Hedgehog Inhibitors (HHi). The lower‐than‐expected efficacy of anti‐PD‐1 antibodies and limited and conflicting data on PD‐L1 expression in BCC have prompted us to characterize the prerequisites for immune response—PD‐L1, dendritic cells (DCs), and HLA expression with immunohistochemistry in different BCC morphological subtypes, namely superficial, nodular, sclerosing/infiltrative, and basosquamous.

**Methods:**

In this retrospective study, a total of 77 tumor samples from 4 BCC subtypes were analyzed by immunohistochemistry. Among the BCC subtypes, we assessed PD‐L1 and CD11c + DCs expression within tumor and stroma, and HLA Class I expression in the tumor compared to the epidermis. Non‐automated quantification of positive tumor cells and peri‐ and intratumoral immune cells was performed by a board‐certified pathologist.

**Results:**

In all BCC subtypes, tumor cells (SOX9 positive) were negative for PD‐L1. PD‐L1 was expressed on intra‐ and peritumoral immune cells, predominantly in basosquamous BCCs, where 80% of the samples showed PD‐L1 positivity in immune cells. DCs were predominantly found in the peritumoral area, mostly in basosquamous BCC, whereas superficial BCCs showed the least DCs. HLA class I expression was downregulated compared to epidermis in most specimens without significant differences among the subtypes.

**Conclusion:**

Our findings support previous reports on the immune‐privileged status of BCC. Contrary to the literature, we could not confirm PD‐L1 expression on BCC cells, but rather on the intra‐ and peritumoral immune cells. Given these results and the literature suggesting a tendency of higher immunoreactivity compared to other BCC subtypes, basosquamous BCC might be a better target for anti‐PD‐1 therapy as opposed to other subtypes.

## 1 Introduction

Basal cell carcinoma (BCC) is the most frequent cancer in humans and accounts for 75% of all non‐melanoma skin cancers [1]. The majority of BCCs have a good prognosis and can be cured with surgical excision or local therapies [1, 2]. However, a small subset of BCCs can progress into locally advanced (laBCC) or metastatic (1%–10% and 0.0028%–0.5% of all BCCs respectively) and are referred to as advanced BCC (aBCC) [3, 4]. aBCCs often require systemic therapies. The most used therapy targets the hedgehog (HH) pathway, one of the main drivers in the pathogenesis of BCC [5]. However, despite high response rates, 16%–37% of hedgehog inhibitors (HHi) treated patients progressed and 29%–38% had to discontinue therapy due to adverse events, indicating that further treatment options are needed [6, 7, 8, 9].

Anti‐PD‐1 antibody cemiplimab was investigated as second‐line therapy in patients with aBCC, who have progressed or are intolerant to HHi [10]. As a UV‐induced tumor, BCCs carry the highest tumor mutational burden (TMB) among cancers [11], and given the correlation of TMB and response to immunotherapy, it was expected to be very sensitive to immune‐checkpoint inhibitors [12]. However, the response rate was 31% in laBCC, and 22% in mBCC [13, 14].

PD‐L1, the primary PD‐1 ligand, is expressed on antigen‐presenting and non‐lymphoid cells. It inhibits T‐cell function to promote immune tolerance [15, 16]. Many cancers upregulate PD‐L1 to evade immune surveillance [17]. Its expression on tumor cells and infiltrating lymphocytes correlates with anti‐PD‐1 therapy response [18]. A small number of studies have reported conflicting results regarding PD‐L1 expression levels in BCC [19, 20, 21] without considering low‐ and high‐risk subtypes and possible predictions on therapeutic response.

The aim of this study was to investigate differences in immunological marker expression between low‐ and high‐risk BCCs, with possible relevance for therapeutic strategies.

## 2 Materials and Methods

### 2.1 Sample Selection

Twenty samples for each subtype (nodular, superficial, infiltrative, and basosquamous) were selected from the archive of the Dermatopathology laboratory, Department of Dermatology, University Hospital Zurich. Only treatment naïve biopsies from patients over 18 years of age without known syndromic disease (e.g., Gorlin‐Goltz, xeroderma pigmentosum etc.) were used. Formalin‐fixed paraffin‐embedded (FFPE) samples from January 2017 on were included to ensure stable antibody staining [22]. Biopsies over 4 mm in diameter and depth were given preference. Samples with scar tissue due to re‐excisions as well as comprising mixed subtypes [23] were excluded from the study. Frozen sections or biopsies retrieved via micrographic surgery (Mohs) were excluded due to uncertain stability of antibody staining and lack of epidermis as internal control for further analysis. The histopathological sections were evaluated by a board‐certified dermatopathologist (I. K.) to confirm the diagnosis and sufficient tumor content. The same procedure was repeated after the FFPE samples were cut and stained. Three superficial BCC showed no remaining tumor content after cutting and staining and were removed from the analysis. The final cohort included 77 samples: 20 of nodular, infiltrative, and basosquamous each, and 17 superficial subtypes. This study was approved by the local Ethics Committee (BASEC_nr 2017‐00688).

### 2.2 Histopathological Diagnostic Criteria for BCC Subtypes

The histopathological classification of BCC subtypes was performed in accordance with the WHO Classification of Skin Tumors [24], integrating consensus criteria from Fernández‐Figueras et al. [25] Basosquamous carcinoma was defined as a malignant epithelial neoplasm exhibiting both basaloid and squamous differentiation within the same tumor, typically with a transition zone between components (Figure 1A,B). The basaloid component showed features consistent with BCC (including basaloid nests with peripheral palisading), while the squamous component demonstrated cytological atypia and keratinization. Tumors were carefully distinguished from keratotic BCC, collision tumors (BCC and SCC), and reactive pseudocarcinomatous squamous proliferations, and were only included as basosquamous carcinoma if clear biphasic differentiation was present [24, 25]. We have adopted the term “infiltrative” BCC from the consensus criteria, representing a unified high‐risk category encompassing infiltrating, morpheaform/sclerosing, and micronodular subtypes (Figure 1C,D). This simplification was based on the rationale that all three subtypes share an infiltrative growth pattern with a higher risk of deep involvement and subclinical extension, and that distinguishing between them showed poor interobserver reproducibility. It is characterized by irregular strands, cords, or small nests of basaloid cells within a variably desmoplastic stroma, often with deep dermal extension, poorly defined margins, and occasional perineural invasion. The presence of any infiltrative component defines this subtype. Nodular BCC is defined as well‐circumscribed basaloid tumor nodules with peripheral clefting and a non‐desmoplastic stroma; it is considered low‐risk (Figure 1E,F). Superficial BCC shows multiple tumor aggregates emerging from the epidermis and confined to the papillary dermis within a loose, myxoid stroma, typically not exceeding 1 mm in depth, and is also classified as low‐risk (Figure 1G,H).

**FIGURE 1:**
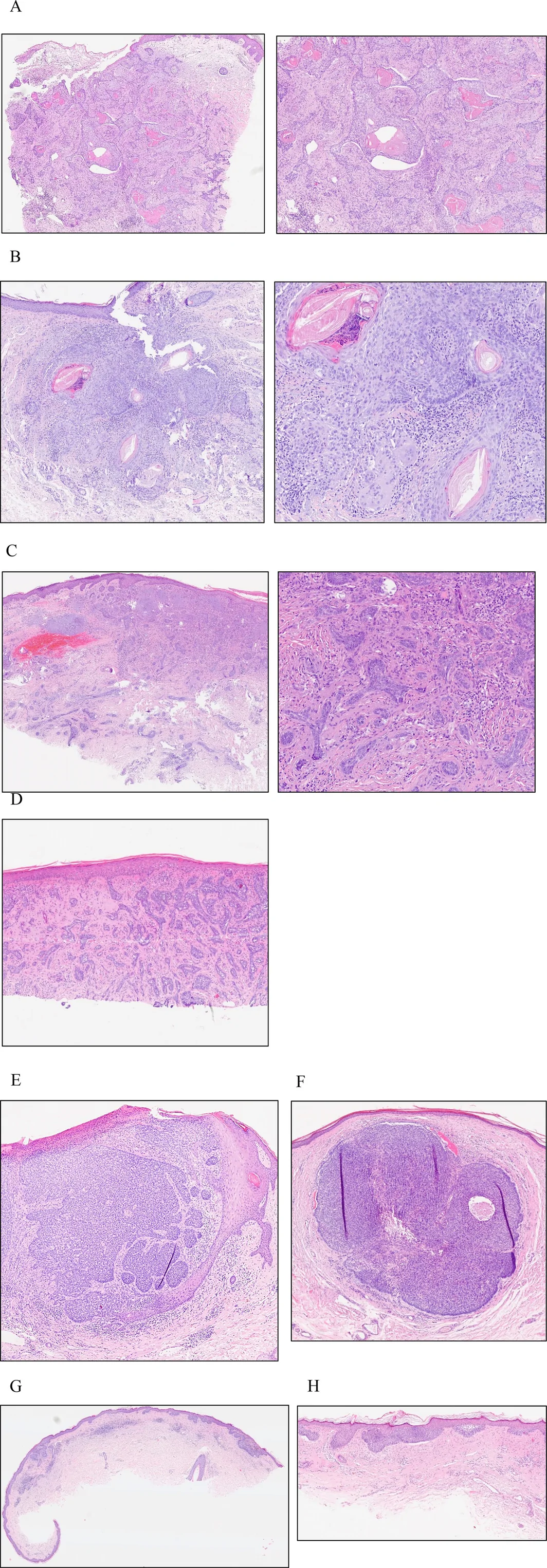
(A–H) *Four BCC subtypes stained with hematoxylin and eosin staining (HE)*. (A, B) Basosquamous BCC exhibiting both basaloid and squamous differentiation with keratinization within the same tumor (A ×20 left, ×40 right; B ×40 left, ×100 right). (C, D) Infiltrative BCC demonstrating irregular strands, cords, or small nests of basaloid cells within a variably desmoplastic stroma (C ×20 left, ×100 right; D ×40). (E, F) Nodular BCC presents well‐circumscribed basaloid tumor nodules with peripheral clefting (E ×20; F ×20). (G, H) Superficial BCC portrays multiple tumor buds descending from the epidermis confined to the papillary dermis (G ×10, H ×20).

### 2.3 Immunohistochemical Staining

Immunohistochemical staining was performed with the LEICA BOND Rx System following the manufacturer's instruction. FFPE sections were cut at 4 μm thickness onto SuperFrost Plus slides and dried by baking at 65°C for 30 min. No manual pre‐treatment steps were required for FFPE sections. Deparaffinization was conducted using Bond Dewax Solution. For HLA class I, the antibody HCA2 was used, and the antigen retrieval was performed using Leica Bond ER2 solution for both antigen retrievals for 30 min at 95°C each (mouse antibody, diluted 1:3000). For the PD‐L1 antibody, antigen retrieval was done using Leica Bond ER2 solution for 20 min at 100°C (rabbit antibody, clone E1L3N, cat.nr. #13684, diluted 1:400). SOX9 was treated with Leica Bond ER2 solution for 30 min at 100°C (rabbit antibody, diluted 1:5000). For antibody staining, the Bond Polymer Refine Red Detection Kit was predominantly used. For the double staining with SOX9 and PD‐L1, the DAB staining of PD‐L1 was performed using the Bond Polymer Refine Detection (DS9800 Leica). After staining, the slides were mounted with a glass coverslip.

### 2.4 Image Analysis and Quantification

Assessment was performed by non‐automatic scoring. We considered that this would ensure higher reliability and validity due to frequent artifacts. A board‐certified pathologist (C. D. S.) additionally assessed each sample.

Each specimen was examined under low magnification to identify areas of PD‐L1 staining on SOX9 positive tumor cells or SOX9 negative peri‐ and intratumoral immune cells. The percentage of PD‐L1 staining was then visually assessed under high magnification. Tumor cells with membranous staining, regardless of the intensity, are considered positive, as opposed to cytoplasmic staining [26]. In contrast, peri‐ and intratumoral immune cells with membranous or cytoplasmic staining are considered positive [27, 28]. A positive PD‐L1 staining on peri‐ or intratumoral immune cells was calculated as a percentage of all immune cells on the respective specimen. Similarly, a positive PD‐L1 staining on tumor cells was calculated as a percentage of all tumor cells on the cut. The percentage of PD‐L1 positivity of cells was estimated by manual scoring. Cases demonstrating at least 1% membranous (cell surface) expression of PD‐L1 were considered positive. We divided the amount of PD‐L1 positive cells into four groups: PD‐L1 positivity in < 1%, 1%–10%, 11%–20%, and 21%–30% (Figures 2A–D and 4A). The categorization of PD‐L1 expression in tumor‐associated immune cells (< 1%, 1%–10%, 11%–20%, 21%–30%) was empirically derived from the observed distribution within our cohort. Across all histological subtypes, the proportion of PD‐L1–positive immune cells ranged from absent to approximately 30% of all immune cells present on the slide. We aimed to reflect this spectrum while preserving clinically and morphologically meaningful distinctions. In particular, cases with sparse immune infiltrates typically corresponded to the 1%–10% category, whereas those with dense infiltrates frequently fell into the 21%–30% range. Introducing an intermediate category (11%–20%) allowed us to better capture these gradations, which would have been insufficiently represented by a simpler three‐tier system. Although visual estimation of percentages may be challenging, semi‐quantitative assessment in approximately 10% increments is considered feasible and reproducible among trained (dermato)pathologists. However, future studies may benefit from automated quantification approaches to further improve precision and standardization.

**FIGURE 2:**
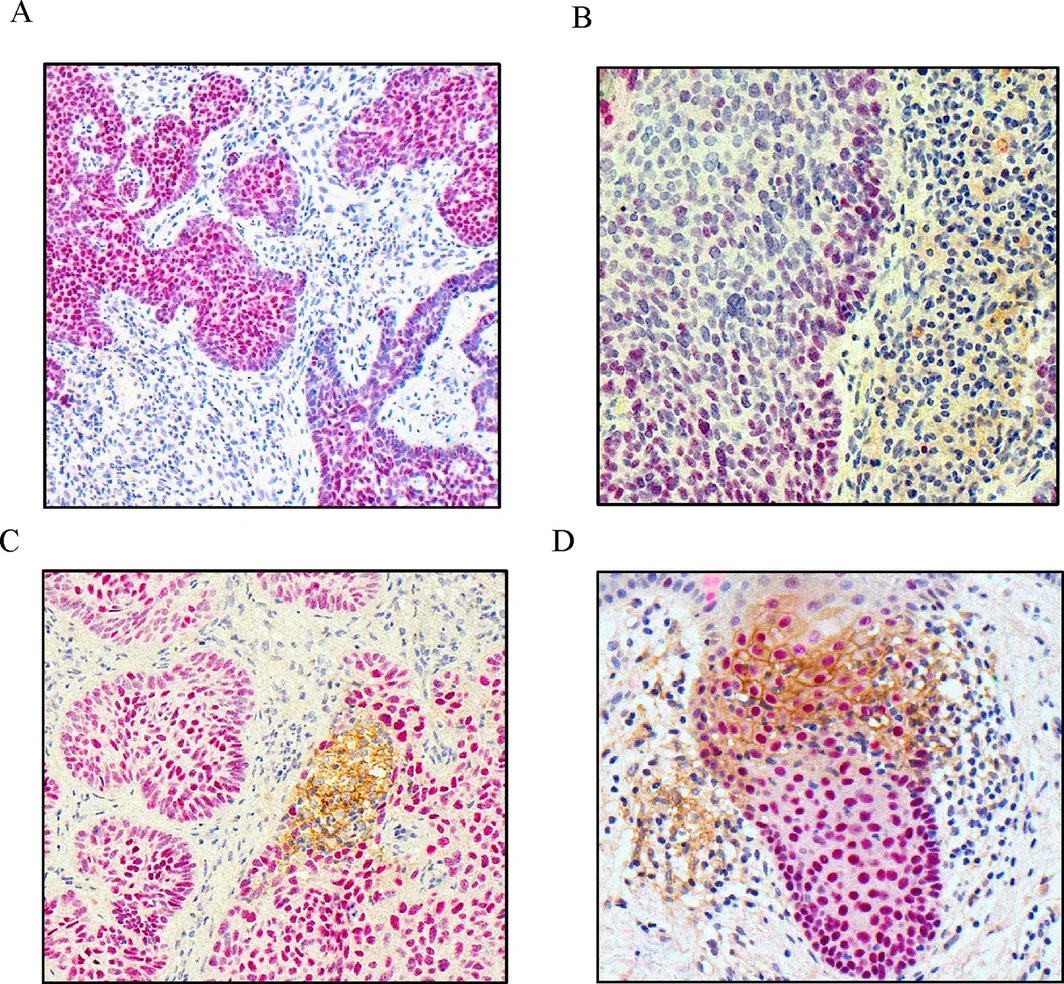
(A–D) *Nodular BCC stained with SOX9 (red) and PD‐L1 (brown) double staining*. (A) Nodular BCC expresses SOX9 and is negative for PD‐L1. This tumor is surrounded by SOX‐9 negative and PD‐L1 negative immune cells; ×100. (B) In this BCC PD‐L1+ immune cells are located peritumorally; ×200. (C) Immune cells expressing PD‐L1 (brown) infiltrate focally into the tumor, thus conferring an apparent PD‐L1 positivity to tumor cells. However, the double staining allows us to assign the PD‐L1 staining exclusively to immune cells, whereas no distinctive membranous PD‐L1 staining in SOX9 expressing BCC is seen; ×200. (D) Inflamed hair follicle: Dual positivity for SOX9 (nuclear red) and PD‐L1 (membranous brown) of follicle infundibular squamous cells in an area with infiltrative immune cells; ×200.

CD11c staining was performed to identify the quantity and location of dendritic cells (DCs) in regard to the tumor (peritumoral versus intratumoral). We categorized the number of DCs into four groups: none, few, moderate, and many (Figures 3A–C and 4D). In addition, the localization of DCs was classified as only peritumoral and peri‐ as well as intratumoral (Figure 4B,C).

**FIGURE 3:**
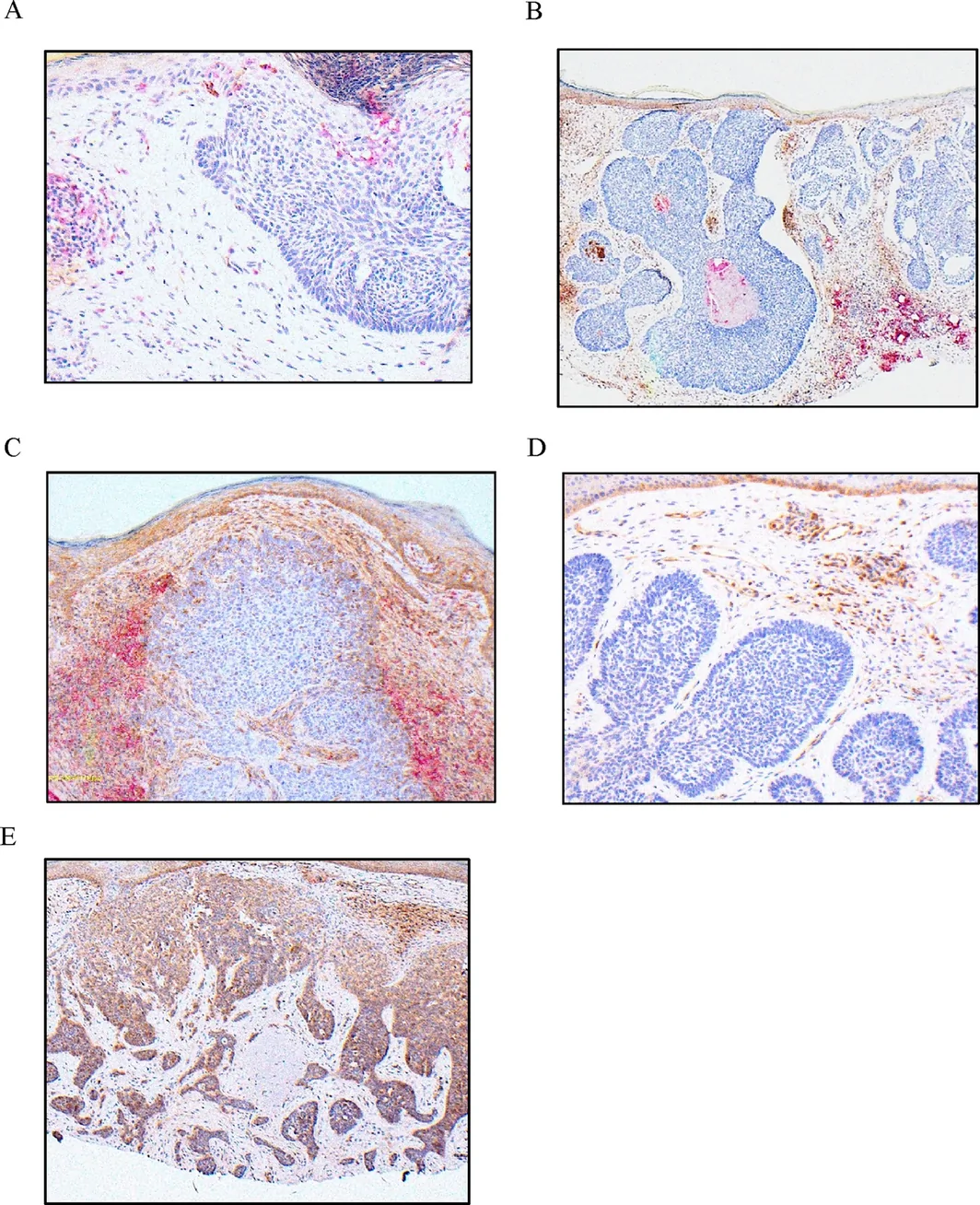
(A–E) Different BCC subtypes stained with CD11c (red) and HLA class I (HCA: Brown) double staining. (A) Few CD11c stained DCs located peri‐ and intratumoral in a nodular BCC; ×100. (B) Moderate amount of CD11c stained DCs located peritumoral in a nodular BCC; ×20. (C) Many CD11c stained DCs located peritumoral in a nodular BCC; ×100. (D) Positive staining (brown) in the epidermis, predominantly in basal cells and negative expression in nodular BCC; no demonstration of CD11c dendritic cells; ×100. (E) Positive HLA I staining in this infiltrative BCC with equal intensity in both epidermis (control) and BCC; ×20.

**FIGURE 4:**
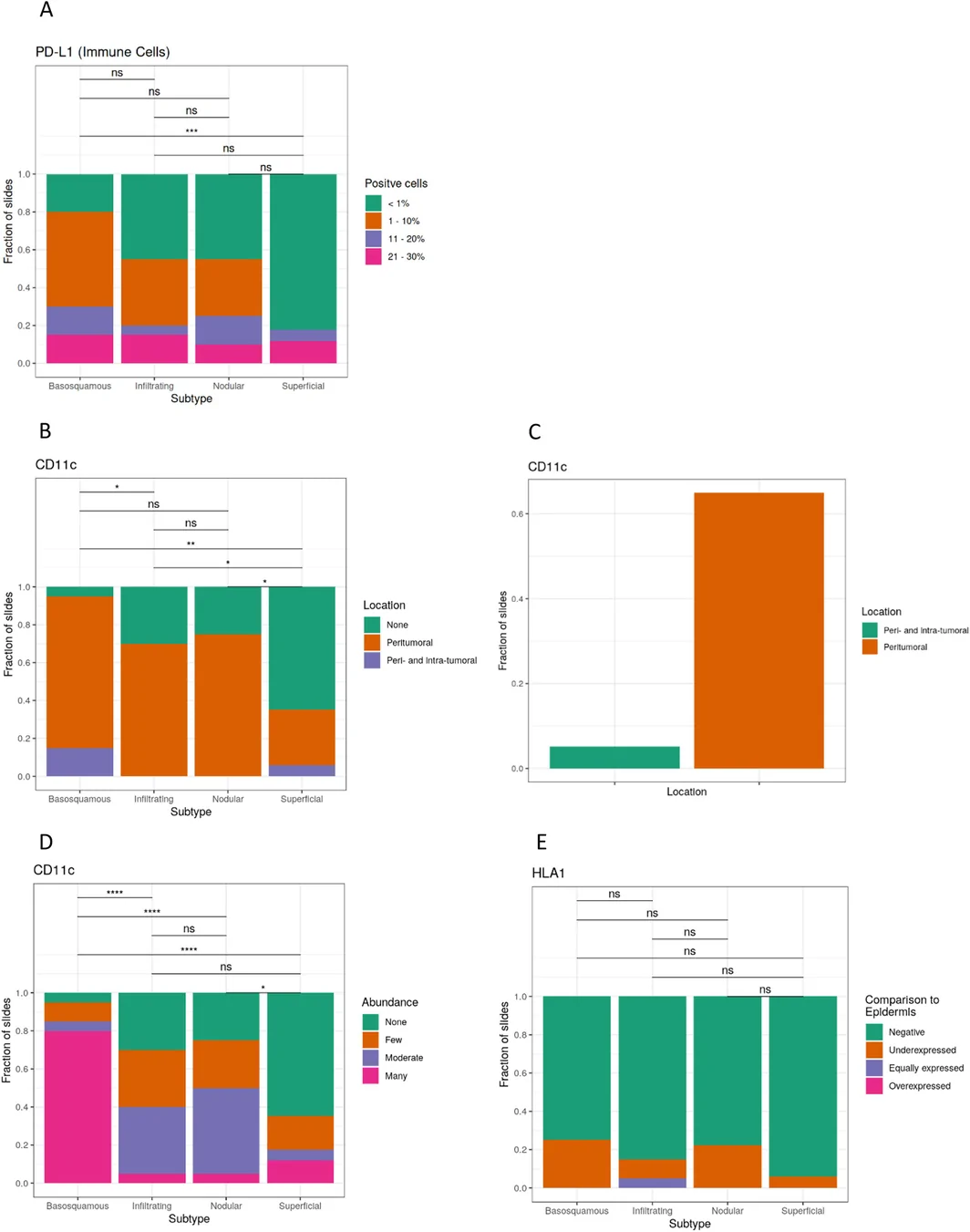
Bar charts with BCC subtypes (A–E) or location of DCs (C) on the *x*‐axis. The *y*‐axis represents the percentage of samples, where 100% corresponds to 20 samples for basosquamous, infiltrative, and nodular subtypes, and 17 samples for the superficial subtype. The height of each colored segment (*y*‐axis) corresponds to 100% of samples for each subtype. *p*‐value symbols: **** < 0.0001; *** < 0.001; ** < 0.01; * < 0.05; ns > 0.05. (A) The number of PD‐L1 positive peri‐ and intratumoral immune cells within BCC subtypes calculated as a percentage of all immune cells on the respective specimen (< 1%, 10%, 20%, and 30%). (B) Location of CD11c + DCs among BCC subtypes: No DCs, peri‐ and intratumoral, versus peritumoral only. (C) Location of CD11c + DCs among all BCCs: Peri‐ and intratumoral, versus peritumoral only. (D) Abundance of CD11c + DCs among BCC subtypes: None, few, moderate and many. (E) Intensity of HLA class I staining on tumor cells, compared to the basal epidermis.

HLA Class I staining within the different BCC subtypes was assessed in comparison to the epidermis as a reference, as the epidermis always expresses HLA Class I [29]. The staining of the tumor cells was divided into four categories: not stained, underexpressed, equally expressed, or overexpressed compared to the epidermis (Figures 3D,E and 4E).

### 2.5 Statistical Method

Within each staining, the numbers of slides falling into each category were compared between subtypes using Fisher's Exact Test. *p*‐values were corrected for multiple testing using the Benjamini–Hochburg correction. All tests were performed in R version 4.4.0.

## 3 Results

### 3.1 PD‐L1, CD11c, and HLA Class I Expression Levels Among BCC Subtypes

*PD‐L1 was not detected on BCC cells of any subtype but was expressed on peri‐ or intratumoral immune cells predominantly in basosquamous, nodular, and infiltrative BCCs*.

None of the BCC subtypes expressed PD‐L1 on the tumor cell surface at a detectable level (> 1%) (Figures 2A–D and 4A). However, immune cells showed PD‐L1 positivity across all BCC subtypes. The highest expression was observed in basosquamous BCC, where 80% of the samples showed any PD‐L1 positivity in immune cells, with 19% of them showing 20% and 30% of PD‐L1+ peritumoral immune cells, while 10% of peritumoral immune cells exhibited PD‐L1 positivity in 63% of the samples.

Infiltrative and nodular BCC subtypes showed PD‐L1+ immune cells in 55% of samples, with a comparable distribution of PD‐L1 expression levels (Figure 4A). In contrast, superficial BCC exhibited the lowest PD‐L1 expression, with only 17.6% of samples displaying PD‐L1 positivity in up to 20% of peri‐ and intratumoral immune cells. The difference in PD‐L1 expression between basosquamous and superficial BCC was statistically significant (*p* < 0.001), while no significant differences were observed among the other subtypes.

*Basosquamous BCCs showed the highest fraction of DCs, while superficial BCC had the lowest. The DCs were mainly located peritumorally and almost never intratumorally*.

Among all samples, 5% had DCs detectable in the peri‐ and intratumoral area, 65% had peritumoral DCs only, and 30% had no DCs (Figures 3A–C and 4B,C). Basosquamous BCCs showed the highest amount of DCs, followed by nodular BCC and infiltrative, while superficial had the least. When looking at the abundance of DCs, basosquamous BCC showed significantly higher abundance than other subtypes (*p*‐value < 0.0001) with many DCs found in 80%. Other subtypes showed predominantly moderate to few cells (Figure 4D). Yet nodular BCC showed a significantly higher abundance than superficial BCC (*p*‐value < 0.05).

*HLA class I is largely downregulated within all BCC subtypes*.

Tumor cells were either negative or showed low expression of HLA Class I compared to epidermis without significant difference among the subtypes. An exception was one infiltrative BCC that showed equal expression (Figures 3D,E and 4E).

## 4 Discussion/Conclusion

BCC is a tumor with a very high mutational burden, yet systemic anti‐PD‐1 therapy demonstrates only moderate clinical efficacy, with response rates not exceeding 31%. Anti‐PD‐1 response has been associated with higher PD‐L1 expression levels in different cancer types. High TMB and PD‐L1 expression in locally advanced BCC, however, could not predict the response to cemiplimab [14].

The literature shows inconsistent data on PD‐L1 expression in BCC. Chang et al. reported PD‐L1 positivity in 89.9% of tumor cells and 94.9% of tumor‐infiltrating lymphocytes using a > 5% threshold [19]. These high rates likely reflect their inclusion of both cytoplasmic and membranous staining in tumor and immune cells. However, most authors consider only membranous staining in tumor cells and either membranous or cytoplasmic staining in immune cells as positive [26, 27, 28, 30]. Other studies reported substantially lower levels: Lipson et al. found PD‐L1 expression in 22% of tumor cells and 82% of lymphocytes [20] and Gompertz‐Mattar et al. reported a mean of 5.4% across 115 BCCs [31]. They looked at differences among nodular, superficial, and infiltrative BCC. PD‐L1 expression was highest in nodular BCC (intratumoral 0.13%; stromal 11.8%) and nearly absent in superficial and morpheaform subtypes. Yülek et al. found no PD‐L1 expression in 42 BCC outside the head and neck region [21]. Altogether, these findings indicate that BCC is generally poorly immunogenic, which may explain the modest response to PD‐1 blockade [32, 33].

Additionally, we assume that the reported inconsistency of PD‐L1 positivity among BCCs may partly result from the morphological difficulty in distinguishing PD‐L1+ immune cells infiltrating or surrounding tumor nests from true PD‐L1 expression on tumor cells. To address this issue, we performed double staining for PD‐L1 and SOX9, the latter serving as a nuclear marker for BCC cells. This approach facilitated a more reliable allocation of PD‐L1 positivity, allowing us to differentiate between immune cells and tumor cells more effectively. Across all histological subtypes, we did not observe any PD‐L1 expression on tumor cells. Instead, PD‐L1 expression was predominantly detected in peritumoral immune cells, most prominently in the basosquamous subtype, followed by the nodular and infiltrative types, and least in superficial BCCs. Only membranous PD‐L1 staining was considered positive in tumor cells, whereas membranous and cytoplasmic PD‐L1 staining in immune cells was scored positive. We also noted that inflamed hair follicles can exhibit membranous PD‐L1 positivity, which must be differentiated from BCCs expressing PD‐L1 (Figure 2D). Therefore, we consider PD‐L1 and SOX9 double staining a valuable approach to minimize misinterpretation of PD‐L1 expression in BCCs.

Basosquamous carcinoma remains a diagnostically and biologically controversial entity, with ongoing debate regarding its classification and its relationship to both BCC and cutaneous squamous cell carcinoma. Histologically, its biphasic differentiation and frequent transition zones support a spectrum rather than a strictly separate category. Notably, the squamous component shares morphological and potentially biological features with cutaneous squamous cell carcinoma, including keratinization and cytological atypia [24]. This overlap may be particularly relevant in the context of immunotherapy. Cemiplimab has demonstrated substantial efficacy in advanced cutaneous squamous cell carcinoma, and it is conceivable that tumors with partial squamous differentiation, such as basosquamous carcinoma, may exhibit treatment responses more similar to SCC than to conventional BCC. However, data on this subgroup remain limited, and further studies are warranted.

In our study, the higher PD‐L1 expression in immune cells within basosquamous BCC suggests greater immunogenicity in this subtype, which may reflect its potential to transdifferentiate into squamous cell carcinoma (SCC), a tumor type with heightened immunoreactivity and increased incidence in immunosuppressed individuals [34, 35]. Also, if PD‐L1 positivity in tumor‐associated immune cells correlates with therapeutic response, as shown in melanoma [36], then basosquamous BCC could represent a more responsive subtype to anti‐PD‐1 therapy. Low‐risk subtypes, on the other hand, might benefit from combination strategies that enhance tumor immunogenicity, such as imiquimod and HHis, both of which increase HLA Class I expression and promote immune cell infiltration [35, 37]. Using CD11c staining, we identified DCs [38]. In BCC, these cells were found to frequently lack T cell co‐stimulatory molecules (CD80/CD86), hence be impaired in antigen presentation and contributing to tumor immune evasion [39]. In our study, CD11c + cells were mostly abundant in the high‐risk basosquamous BCC, and located in peritumoral stroma, with minimal infiltration into the tumor nests. These cells are most likely functionally impaired which could explain the invasive characteristics in this subtype. Another high‐risk BCC subtype—infiltrative BCC—was reported to exhibit lower numbers of functional DCs [40]. The relevance of this remains unclear and requires further characterization of the cell phenotype and functional status.

We also confirmed that HLA Class I expression is broadly downregulated across all subtypes, consistent with previous reports [35]. The same pattern was also observed in basosquamous subtypes, apart from a single sample, which showed equally strong HLA Class I expression as the epidermis. HLA Class I downregulation, along with an abundance of regulatory T cells, is considered a key immune escape mechanism in BCC [41].

The strength of our study lies in the strict inclusion of pure BCC subtypes, allowing for a more accurate assessment of subtype‐specific immune profiles. The histopathological diagnosis for the BCC subtypes was performed according to the WHO classification of skin tumors, while integrating the European Consensus Project [24, 25]. Fernandez‐Figueras et al. employed a modified Delphi method among 12 recognized dermatopathologists from nine European countries to establish consensus definitions for BCC subtypes. The project initially used the WHO 2018 Classification of Skin Tumors definitions and subsequently proposed a simplified classification with improved interobserver reproducibility.

However, PD‐L1 immunohistochemistry remains a technically challenging procedure. Variability in antibody clones (e.g., 22C3, SP263, SP142, 28‐8), staining platforms, and scoring systems hinder standardization and comparability across studies [42, 43]. Our findings emphasize the importance of standardized methodology and strict histopathological classification in evaluating immune biomarkers in BCC. The semi‐quantitative categorization of PD‐L1–positive tumor‐associated immune cells described in the Methods section is feasible for trained pathologists using visual 10% increments, although automated quantification may further enhance reproducibility.

In conclusion, our data suggest that the immunological landscape of BCC varies by subtype. Basosquamous BCC displays features of a relatively more immunogenic tumor microenvironment and may be more amenable to PD‐1 blockade. These insights support the rationale for subtype‐driven immunotherapeutic strategies and underscore the need for further investigation into the functional role of immune cells in BCC.

## Author Contributions

Reinhard Dummer, Gaetana Restivo, Egle Ramelyte, Isabella Dommann, and Isabel Kolm contributed to the conceptualization of the study. The methodology was set up by Reinhard Dummer, Isabella Dommann, Gaetana Restivo, Egle Ramelyte, Federica Sella, and Isabel Kolm. The validation was performed by Isabel Kolm, Corina Dommann‐Scherrer, and Gaetana Restivo; Patrik Turko and Isabella Dommann conducted the formal analysis; Isabella Dommann and Federica Sella performed the investigation; the resources were provided by Reinhard Dummer, Mitchell Levesque, and Soldano Ferrone; the original draft preparation was performed by Isabella Dommann, Gaetana Restivo, and Egle Ramelyte; Soldano Ferrone, Isabel Kolm, Gaetana Restivo, Egle Ramelyte, Isabella Dommann, Phil Cheng, Patrick Turko, Reinhard Dummer, and Mitchell Levesque contributed to the review and editing; visualisation of the data was performed by Isabella Dommann, Patrick Turko, Gaetana Restivo, and Phil Cheng; Gaetana Restivo, Egle Ramelyte, and Reinhard Dummer supervised the study; project administration was conducted by Reinhard Dummer, Gaetana Restivo, and Egle Ramelyte; All authors have read and agreed to the published version of the manuscript.

## Funding

The authors have nothing to report.

## Ethics Statement

Study approval statement: The study was conducted in accordance with the Declaration of Helsinki, and approved by the local (Ethics Committee) of the Canton of Zürich (BASEC nr 00688 approved on 22.06.2017).

## Consent

Written informed consent was obtained from all subjects involved in this study.

## Conflicts of Interest

The authors declare no conflicts of interest.

## Data Availability

The data that support the findings of this study are available from the corresponding author upon reasonable request.
